# Characterization and feature selection of volatile metabolites in Yangxian pigmented rice varieties through GC-MS and machine learning algorithms

**DOI:** 10.3389/fnut.2025.1598875

**Published:** 2025-05-20

**Authors:** Kaiqi Cheng, Ruonan Dong, Fei Pan, Wen Su, Lingjie Xi, Meng Zhang, Jingzhang Geng, Ruichang Gao, Wengang Jin, A. M. Abd El-Aty

**Affiliations:** ^1^Qinba State Key Laboratory of Biological Resources and Ecological Environment, QinLing-Bashan Moun-tains Bioresources Comprehensive Development 2011 C. I. C, Shaanxi Province Key Laboratory of Bio-Resources, College of Bioscience and Bioengineering Shaanxi University of Technology, Hanzhong, China; ^2^Institute of Apicultural Research, Chinese Academy of Agricultural Sciences, Beijing, China; ^3^College of Food and Biotechnology, Jiangsu University, Zhenjiang, China; ^4^Department of Pharmacology, Faculty of Veterinary Medicine, Cairo University, Giza, Egypt; ^5^Department of Medical Pharmacology, Medical Faculty, Ataturk University, Erzurum, Türkiye

**Keywords:** pigmented rice, metabolites, multivariate statistics, machine learning, volatiles

## Abstract

**Introduction:**

Pigmented rice is fascinated by consumers for its abundant phytochemicals and unique aroma.

**Methods:**

In this study, GC–MS-based metabolomics of Yangxian colored rice varieties were performed to characterize their volatile metabolites through multivariate statistics and machine learning algorithms.

**Results:**

Results showed that a total of 357 volatile metabolites were detected and segmented into 9 groups, including 96 organooxygen compounds (26.89%), 52 carboxylic acids and derivatives (14.57%), 42 fatty acyls (11.76%), 16 benzene and substituted derivatives (4.48%), and 11 hydroxy acids and derivatives (3.08%). Multivariate statistics screened 127 differentially abundant metabolites via PLS-DA. Principal component analysis revealed that the percentages of PC1 and PC2 were 52.48% and 27.09%, respectively. Based on differential metabolites with great multicollinearity above 0.8 and the chi-square test (20% feature numbers), only 7 metabolites were found to represent the overall metabolites among the several colored rice varieties. Four machine learning models were further used for the classification of various colored rice varieties, and random forest model was the optimum for predicting classification, with an accuracy of 0.97. Moreover, Shapley additive explanations analysis revealed that the 7 metabolites can be used as potential markers for representing the metabolomic profiles.

**Conclusions:**

These results implied that GC–MS-based metabolomics combined with random forest might be effective for extracting key features among different pigmented rice varieties.

## 1 Introduction

Pigmented rice is a popular and healthy cereal fascinated by consumers because of the abundance of phytochemicals, which promote favorable health benefits (1). The primary foundation of skin coloration in rice is anthocyanins, which are flavonoids (2). In recent years, pigmented rice has garnered increasing attention for its abundance of bioactive compounds, particularly anthocyanins and flavonoids. These phytochemicals are known for their potent antioxidant and anti-inflammatory properties. A recent study by Callcott et al. (3) demonstrated that acute consumption of purple and red rice significantly improved plasma antioxidant capacity and reduced levels of proinflammatory cytokines in obese individuals. In parallel, global market trends have shown a steady rise in consumer demand for functional rice products (4). In addition, volatile flavors are also highly important for the assessment of rice quality because they can be used to determine the quality grade and price of rice in retail markets (5). Yangxian County (Hanzhong, China) is renowned for its native-colored rice provisions. Yangxian pigmented rice is mainly black, yellow, purple, red, or green in color and contains abundant fibers and bioactive components (6, 7). Therefore, pigmented rice has great potential for use in many applications and needs extra attention.

Currently, metabolomics has become an important topic of systems biology after genomics, transcriptomics, and proteomics (8). Metabolomics could provide complete information for observing the variations in low-molecular-weight chemicals in samples, and is targeted at revealing the relative changes among physiopathological variations, and processing circumstances (9). GC-MS is one of the most widely utilized approaches in metabolomic analysis, because of its low cost, repeatability, and simple data statistics, which are different from those of LC-MS or NMR (10, 11).

Although volatile organic compounds detected by GC-IMS and GC-MS methods can be used to discriminate the volatile compounds of various colored rice varieties, they are not sensitive enough to measure smaller variations in the same rice (12). To overcome this disadvantage, volatile metabolites have been a concern during the past decade. TianXin et al. (13) performed a metabolomic assay on rice from several different growing locations through GC-MS. Zhang et al. (14) compared the distinctive abundant metabolites among colored rice and white rice via a broadly targeted metabolomics method. Wang et al. (15) also revealed the traits of distinct odor chemicals in oats through broadly targeted GC–MS metabolomic techniques. These GC-MS-based metabolomic studies can acquire useful information and large amounts of data, which are often processed through multivariable statistics and contain redundant information. With the rapid development of artificial intelligence, machine learning technologies (decision trees, longistic regression, competitive adaptive reweighed sampling, etc.) have been applied during metabolomic studies; these methods can overcome these shortcomings to a great extent and exhibit great application prospects. For example, using machine learning, Zheng et al. (16) demonstrated the valid identification of more informative features from sophisticated metabolomic data on metabolites of honey and sugar in diets fed to mice. Metabolomics coupled with machine learning procedures for classifying the production regions or geographic origins of tea samples has also been published (17, 18).

Our previous studies explored the odor substances of five different colored rice varieties (raw, cooked, and puffed) (6, 9) and performed anthocyanin quantitative analysis, and health-promoting functions in Yangxian (19). However, the results of the metabolomic profiling of five different pigmented rice varieties are still unknown. In this study, differentially abundant metabolites in several Yangxian colored rice varieties were first investigated via GC-MS-based metabonomics combined with multivariate statistical analysis. Moreover, four machine learning approaches were also employed to extract the key significant features of the differentially abundant metabolites in different pigmented rice varieties, and their robustness for the discrimination of pigmented rice was also evaluated, with the hope of shedding additional light on the quality characteristics of Yangxian pigmented rice.

## 2 Materials and methods

### 2.1 Materials

**Figure 1:**
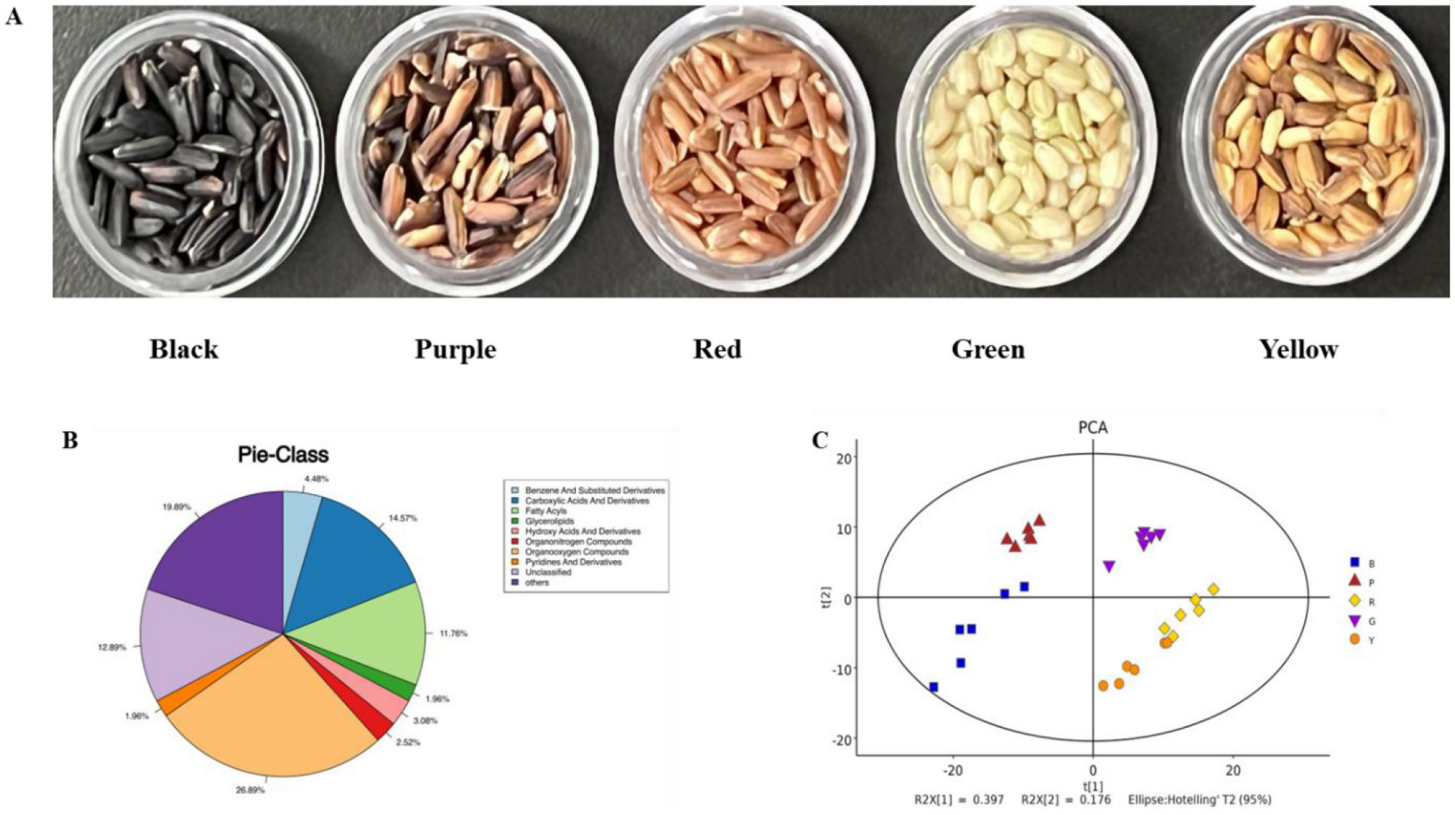
Volatile metabolomic profiles of different pigmented rice varieties. Photos of different pigmented rice varieties used **(A)**, pie-class plot of overall metabolites in different pigmented rice varieties **(B)**, and PCA score plot of overall metabolites in different pigmented rice varieties **(C)**.

The five colored rice varieties were grown and collected in Yangxian, which was provided by Shuangya Zhoudahei Organic Food Co., Ltd (Hanzhong, China). The varieties used were Shuangya Black (moisture content of 11.77 ± 0.08 g/100 g, starch content of 73.04 ± 0.18 g/100 g, crude protein content of 10.68 ± 0.14 g/100 g, lipid content of 2.96 ± 0.05 g/100 g, ash content of 1.56 ± 0.05 g/100 g), Shuangya Green (moisture content of 12.45 ± 0.11 g/100 g, starch content of 73.15 ± 0.19 g/100 g, crude protein content of 9.54 ± 0.11 g/100 g, lipid content of 3.49 ± 0.02 g/100 g, ash content of 1.37 ± 0.03 g/100 g), Shuangya Purple (moisture content of 11.95 ± 0.11 g/100 g, starch content of 74.82 ± 0.18 g/100 g, crude protein content of 9.12 ± 0.12 g/100 g, lipid content of 2.76 ± 0.08 g/100 g, ash content of 1.35 ± 0.06 g/100 g), Shuangya Red (moisture content of 11.57 ± 0.08 g/100 g, starch content of 74.99 ± 0.10 g/100 g, crude protein content of 9.76 ± 0.09 g/100 g, lipid content of 2.44 ± 0.03 g/100 g, ash content of 1.25 ± 0.05 g/100 g), and Shuangya Yellow (moisture content of 11.55 ± 0.08 g/100 g, starch content of 73.52 ± 0.10 g/100 g, crude protein content of 10.23 ± 0.06 g/100 g, lipid content of 3.11 ± 0.03 g/100 g, ash content of 1.58 ± 0.05 g/100 g) (20). The appearance photo of these colored rice was shown in Figure 1A.

### 2.2 Rice pretreatment and extraction

The pigmented rice was processed and extracted according to a modified procedure from Wang et al. (15). Thirty milligrams of tissue sample was accurately weighed into a 1.5 mL vial, and 20 μL of inner reference solution (0.3 mg/mL L-2-chlorophenylalanine in methanol) and 600 μL of methanol-water (4:1, v/v) solution were added. Two tiny steel balls were added, placed in a −80°C environment for 2 min, ground in a grinder, mixed with 120 μL of chloroform, vortexed for 2 min, placed in an ice water bath, subjected to ultrasonication at 40 kHz for 10 min, and left at −20°C for 30 min. After centrifugation at 4°C and 13,000 r/min for 10 min, 100 μL of the upper liquid was pooled and placed into a derived bottle. Quality control (QC) was performed by blending the same quantity of the sample extract solutions. The QC volume was consistent with that of the sample. A centrifugal concentration desiccator was used to evaporate the sample to dryness, and the sample was transferred to a glass derivatization vial. Then, the oxime reaction was performed. The sample was removed, 50 μL of BSTFA (1% trimethyl chlorosilane) original chemical mixture, 20 μL of n-hexane, and 10 μL of inside references (11 fatty acid methyl esters in chloroform) were added, and the mixture was vortexed. The sample was subsequently removed and kept at 25°C for 30 min for subsequent GC–MS metabolomic measurements. Different pigmented rice samples (6 biological replicates each) were labeled according to their outer pigment, namely, black (B), purple (P), red (R), green (G), or yellow (Y).

### 2.3 GC–MS metabolomic assay

The chromatographic equipment used was a DB-5MS capillary column (30 m × 0.25 mm, 0.25 μm, Agilent Technologies, Inc., USA); the transport gas was helium (purity ≥99.99%), the flow rate was 1.2 mL/min; 300°C; 1 μL; the solvent delay was 5 min; the programmed temperature increase was 60°C, the temperature was maintained for 0.5 min; the temperature was elevated to 125°C at 8°C/min, and the temperature was held for 5 min; the temperature was elevated to 210°C at 5°C/min and held for 5 min; the transmission line temperature was elevated to 270°C at 10°C/min and held for 5 min; the temperature was elevated to 305°C at 20°C/min and held for 5 min; the mass spectrometry conditions: the electron ionization source was set at 330°C; and the transmission line temperature was 280°C (21).

### 2.4 Identification and enrichment assay of differentially abundant metabolites

To analyze the distinctness of various sample groups, a PLS-DA model with VIP distribution by SIMCA 14.1 was used. To avoid overfitting, a displacement test using 200 permutations was also carried out. The sieved differentially abundant metabolites (VIP ≥ 1, *P* < 0.05) were identified through the online KEGG database.

### 2.5 Machine learning approaches and simulation assessment

Decreasing the number of features hinders dimensional issues, improves model generalization and decreases overfitting. Briefly, the correlation coefficients of differentially abundant metabolites (features) were evaluated, and metabolites with great multicollinearity (above 0.8) were deleted (diminishing the features from 127 to 37). Furthermore, 20% of the features were processed via the chi-square test (diminishing the features from 37 to 7) (16). Four machine learning algorithms (XG Boost, random forest, decision tree, and longistic regression) were employed to characterize the key metabolomic profiles of different pigmented rice varieties. XGBoost was selected for its high accuracy and ability to capture complex feature interactions, whereas logistic regression offers a simple and interpretable baseline. Random forest provides robustness against overfitting in high-dimensional data, and decision trees offer a clear, interpretable classification structure (22). These algorithms were executed via the Python package (https://anaconda.org/anaconda/conda). Moreover, StratifiedKFold was carried out on the basis of the ratio of positive and negative samples, and 3-fold cross-validation was implemented, through the optimum models for observing the key metabolites among different pigmented rice varieties. After training each model, metrics such as precision, recall, accuracy, and the f1 score were calculated to evaluate the robustness of the models, as illustrated by the following equations:


$$\begin{array}{l} \text{Precision}=\frac{\text{True Positives}}{\text{True Positives + False Positives}}\\ \text{ Recall}=\frac{\text{True Positives}}{\text{True Positives + False Negatives}}\\ \text{Accuracy}=\frac{\text{True Positives + True Negatives}}{\begin{array}{l} \text{True Positives + True Negatives +}\\ \text{False Positives+ False Negatives} \end{array}}\\ \text{ F1 Score}=\text{2}\times \frac{\text{Precision}\times \text{Recall}}{\text{Precision + Recall}} \end{array}$$

### 2.6 Shapley additive explanations (SHAP) analysis

The SHAP analysis is a “model interpretation” procedure derived from Python that explains the output of machine learning algorithms. In SHAP, an extra interpretation model is built, and all features are deemed “contributors”. For every forecast, the model yields a forecast value, which can show the effect of the key features on different pigmented rice varieties and demonstrate the impact outcome (23).

## 3 Results and discussion

### 3.1 Outlook of metabolites in different colored rice varieties

To demonstrate the overall metabolomic profiles, the QC spectra and detailed metabolites in different pigmented rice samples obtained via GC-MS analysis are shown in Supplementary Figure S1. The instrumental conditions and spectra confirmed the reliability of the present results from the QC and pigmented rice samples (Supplementary Figure S1). A total of 357 metabolites were measured and segmented into 9 groups according to their chemical traits, including 96 organooxygen compounds (26.89%), 52 carboxylic acids and derivatives (14.57%), 42 fatty acyls (11.76%), 16 benzene and substituted derivatives (4.48%), and 11 hydroxy acids and derivatives (3.08%) (Figure 1B). These findings implied that organooxygen substances, carboxylic acids and derivatives, and fatty acyls were the dominant metabolites in the five pigmented rice varieties, which likely determined their unique aroma profiles.

To depict the metabolic variations identified in the five colored rice varieties, the 357 metabolites detected were analyzed via PCA. The PC1 and PC2 accounted for 39.7 and 17.6%, respectively, of the variance among these colored rice samples (Figure 1C). There was a slight overlap between red-colored rice and yellow-colored rice, and differently colored rice still relatively clustered into different groups. Moreover, hierarchical cluster analysis of the 357 metabolites was also performed, indicating that the metabolites of various colored rice varieties varied to some extent (Supplementary Figure S2). Several studies have shown that variations in metabolic patterns may be associated with genetics, varieties, nutrients, and geographical differences (13, 15). Overall, these data confirmed the high degree of similarity and high dependability among the duplicates. The distinct metabolic profiles of the five pigmented rice varieties were likely due to their genotypes and varieties.

### 3.2 Differentially abundant metabolites in pigmented rice

**Figure 2:**
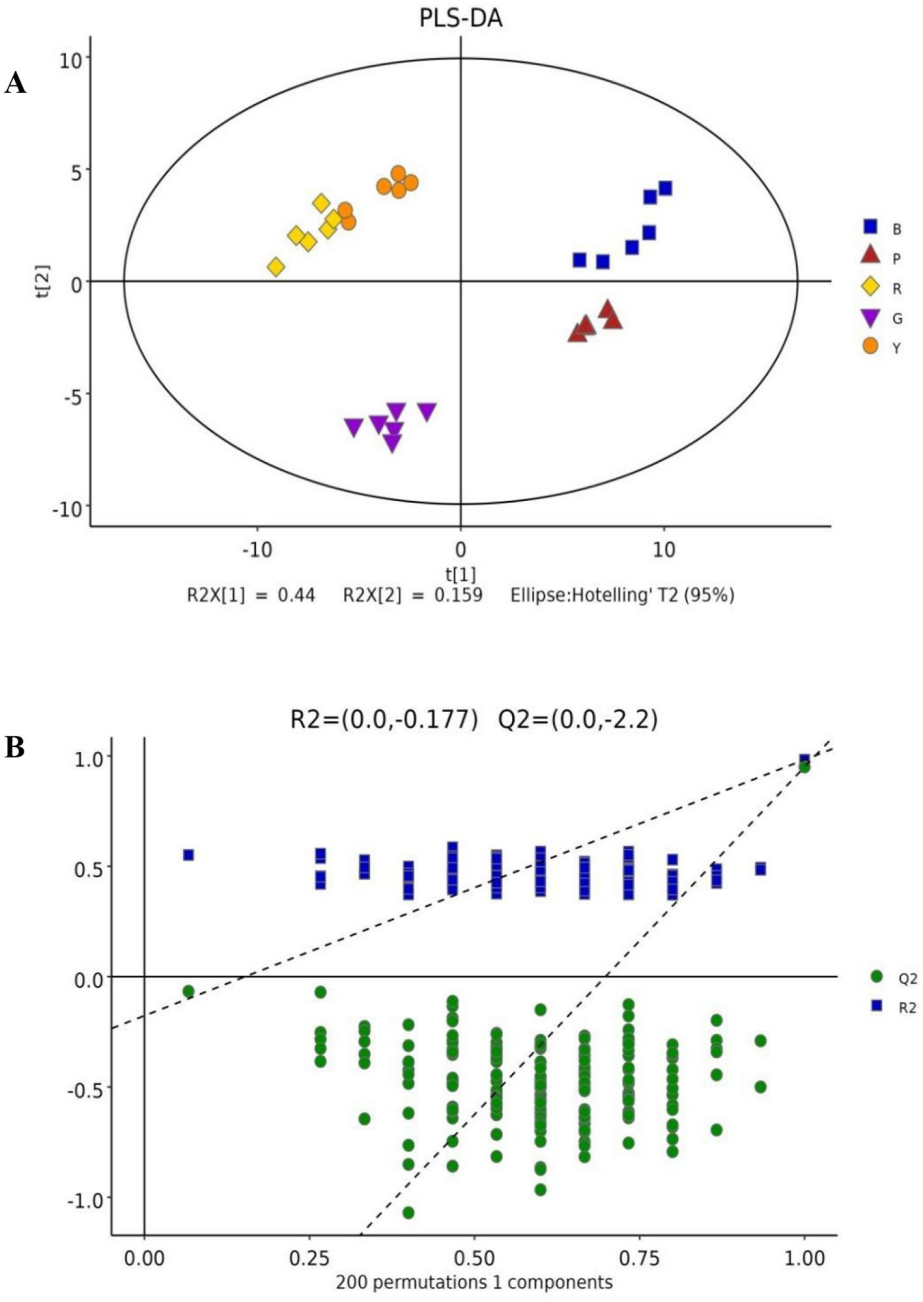
PLS-DA score plot **(A)** and cross-validation by a permutation test **(B)** of the overall metabolites in different pigmented rice varieties.

**Figure 3:**
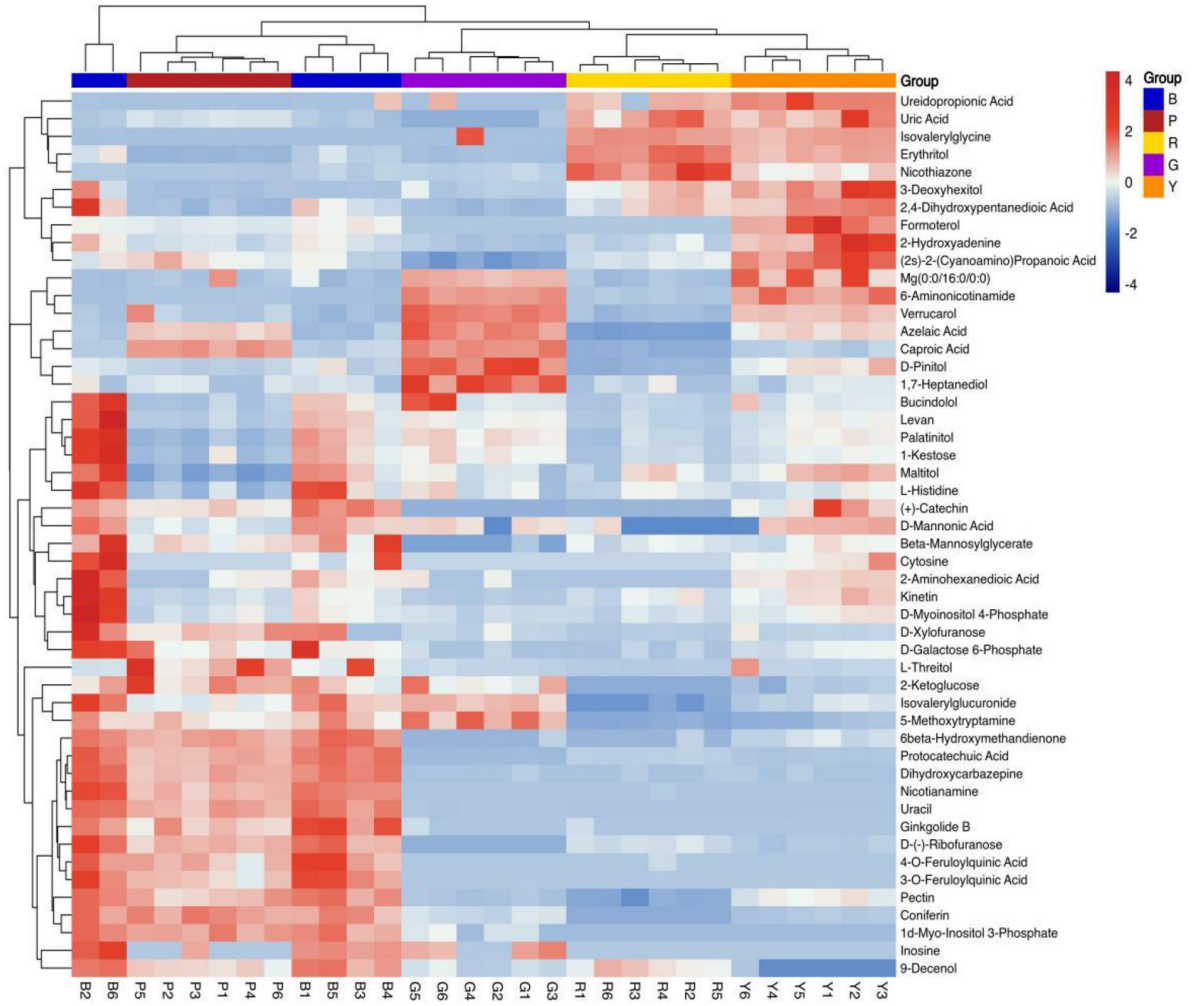
Clustering heatmap of the top 50 differentially abundant metabolites in different pigmented rice varieties.

PLS-DA is frequently utilized to construct interplay equations between variables and sample categories. In the present model, all metabolites identified in several colored rice varieties were fitted via simulation, with *R*^2^*X* (cum) = 0.781, *R*^2^*Y* (cum) = 0.983, and *Q*^2^ (cum) = 0.95. The majority of pigmented rice samples with various colors can be favorably classified on the PLS-DA illustration (Figure 2A), and the discrimination pattern was consistent with the PCA profile (Figure 1C). To elude over-fitting, the dependability of the PLS-DA model was validated by a permutation test, as demonstrated in Figure 2B. After 200 cross-validations, the restoration curve of simulation *Q*^2^ crosses the abscissa, and the intercept is negative (−2.2). In all the permutation tests, *R*^2^ and *Q*^2^ are lower than the original values, implying that the imitation is not overfit (24). The effect of each variable for classification was further evaluated through VIP of the PLS-DA model. On the basis of VIP > 1.0 and a significance level of *p* < 0.05, a total of 127 distinctly abundant chemicals were sieved out among five different pigmented rice samples, which were further subjected to PCA and heatmap clustering analysis. The majority of the variation in pigmented rice might be distinguished via PCA, with a cumulative percentage of 79.57% (the two components were 52.48 and 27.09%, respectively) (Supplementary Figure S3). The heatmap clustering results of the top 50 metabolites in the different pigmented rice varieties are illustrated in Figure 3. As shown, the differentially abundant metabolites in black-colored rice and purple-colored rice were more similar, as both contained higher contents of protocatechuic acid, dihydroxycarbazepine, nicotianamine, uracil, etc. Similarly, black-colored rice had relatively higher contents of levan, palatinitol, maltitol, etc., than purple-colored rice. The characteristic metabolites in green-colored rice are caproic acid, pinitol, 1,7-heptanediol, etc., which are significantly distinct from those in other pigmented rice. Red- and yellow-colored rice shared the majority of the differentially abundant metabolites, but yellow-colored rice contained more metabolites, such as formoterol, 2-hydroxyadenine, and 6-aminonicotinamide (Figure 3). Ch et al. (25) used an HS-GC–MS approach for investigating metabolomic substances to classify rice samples from several states. The present results indicate that the 127 differentially abundant metabolites can also be used for classifying the overall metabolic profiles of various colored rice varieties.

### 3.3 KEGG and enrichment assays of distinctly abundant metabolites

**Figure 4:**
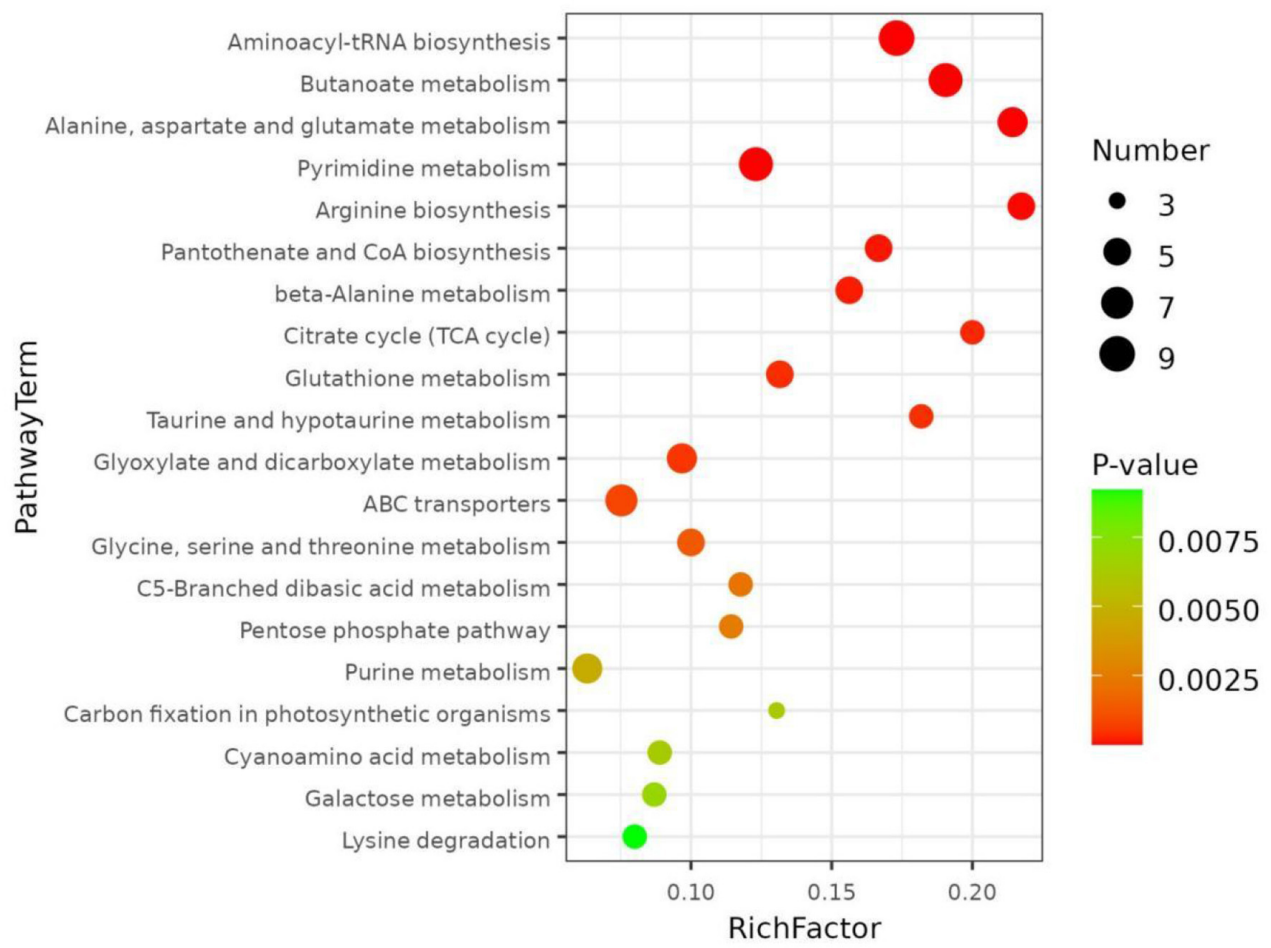
KEGG pathways enriched based on differentially abundant metabolites in different pigmented rice varieties.

The distinct variations in metabolites are closely connected to the metabolic pathways in which they are located. The metabolic pathways that contributed the most to the differentially abundant metabolites among the different pigmented rice varieties were investigated. The KEGG database was utilized to discuss the metabolic pathway accumulation of the distinctly abundant chemicals identified in the present investigation (Figure 4). As shown, the top 3 pathways with significant differences (*p* < 0.05) were aminoacyl-tRNA biosynthesis, butanoate metabolism, and alanine, aspartate and glutamate metabolism in the different pigmented rice varieties.

Aminoacyl-tRNA biosynthesis is chiefly triggered by the direct association of an amino acid with the leading tRNA by a synthetase. Its function is to accurately target amino acids with tRNAs (26). Butanoate metabolism is a vital metabolic pathway under mild salinity stress (27). Alanine, aspartate and glutamate metabolism are in charge of osmotic balance regulation in plants. In addition, glutamate and glutamine produced by these metabolic pathways are imperative factors of phytochelatin (28). Liu et al. (29) compared pathways of differentially abundant metabolites between the normal and colored rice samples and detected alanine, aspartate and glutamate metabolism. Sew et al. (30) reported that pathways associated with the biosynthesis of aminoacyl-tRNA synthesis were significantly enriched in black, red, and white rice groups. The present data were almost similar to those of the previous publications.

### 3.4 Prediction of differentially abundant metabolites via machine learning

As 127 differentially abundant metabolites were much more abundant than the number of different pigmented rice samples (16), they still contained many redundant features, and there was still great potential for overfitting the data, leading to incorrect forecasts (16). Therefore, we processed the data, the correlation coefficients of differentially abundant metabolites (127 features) were evaluated, and metabolites with great multicollinearity (above 0.8) were deleted (diminishing the features from 127 to 37). Moreover, 20% of the features were processed through the chi-square test (diminishing the features from 37 to 7) (Supplementary Figure S4). Similar feature extraction procedures and methods were also published previously (16, 23).

**Figure 5:**
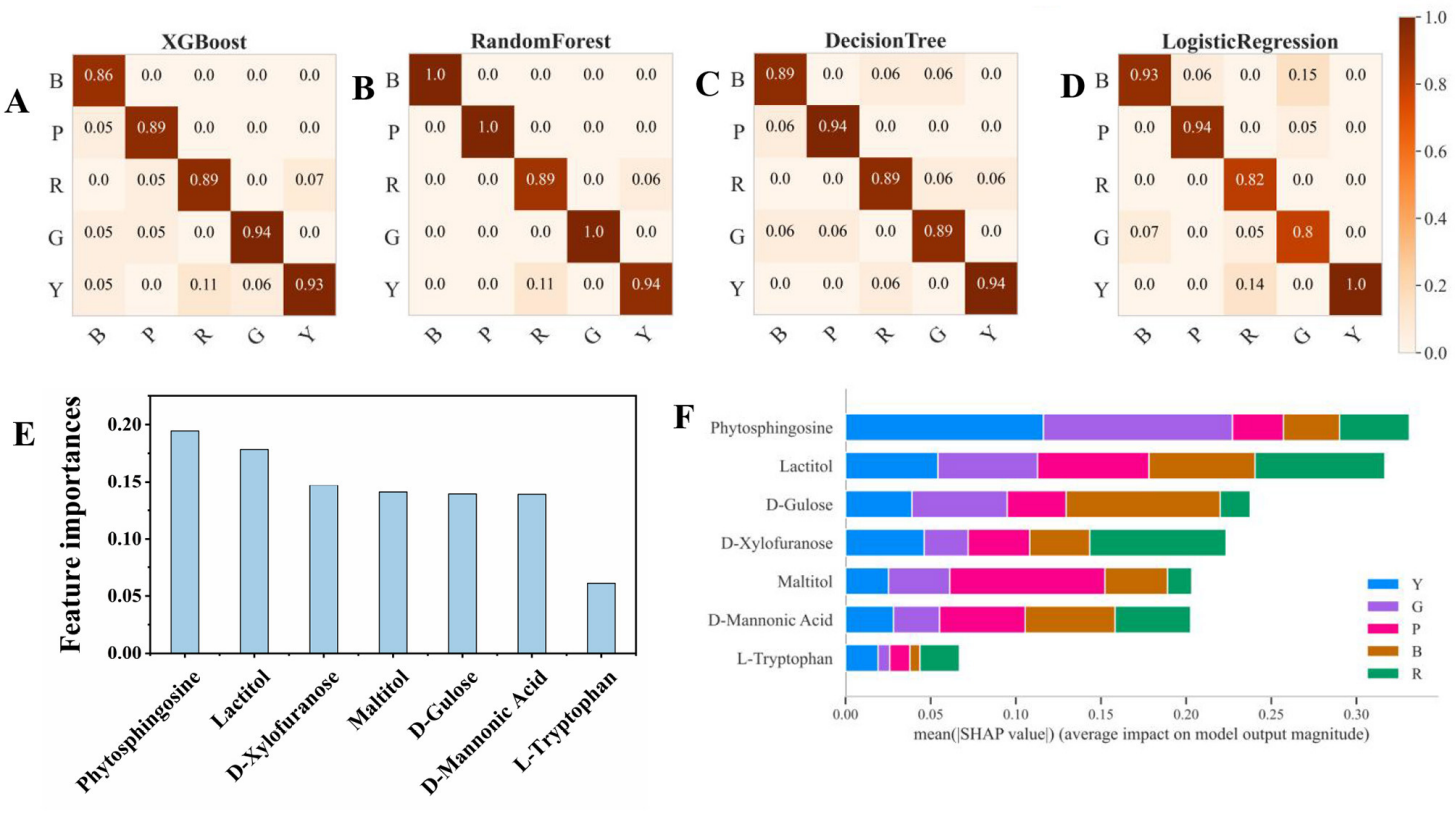
Confusion matrix and feature importance of the 7 metabolites according to the four machine learning models. **(A)** XGBoost, **(B)** random forest, **(C)** decision tree, **(D)** logistic regression, **(E)** feature importance, and **(F)** SHAP values based on random forest.

Four machine learning approaches (XG Boost, random forest, decision tree, and logistic regression) were chosen to mine the data in the present work. XGBoost and random forest, as ensemble methods, are known for their high accuracy and robustness. The decision tree offers simplicity and interpretability, whereas logistic regression provides a solid baseline for binary classification tasks (23). Figures 5A–D shows the four selected machine learning models for predicting the classification of different pigmented rice varieties, and they all show better classification effects with the help of the 7 feature metabolites. Compared with the other models, the random forest model was the best (Figure 5B). Furthermore, the metrics of the four machine learning approaches via the three-fold cross-validation procedure were also evaluated, and the results are shown in Table 1. The outcomes for four assessment criteria, precision, recall, F1 score, and accuracy, suggested that the random forest algorithm scored the highest, with an accuracy of 0.97. Logistic regression displayed the minimum accuracy of 0.89, whereas the accuracies of the XB boost (0.90) and decision tree (0.91) models were comparable (Table 1). In general, the random forest model was the optimum for predicting metabolites among different pigmented rice varieties. Several studies have also shown that a random forest can diminish overfitting and has the best separation, as it introduces randomness, possesses low noise and is applicable for complex data (16).

**Table 1:** Metric parameters of the four machine learning approaches for the discrimination of metabolites.

**Model**	**Precision**	**Recall**	**F1-score**	**Accuracy**
XG boost	0.902	0.900	0.898	0.90
Random forest	0.966	0.966	0.966	0.97
Logistic regression	0.898	0.888	0.888	0.89
Decision tree	0.91	0.91	0.91	0.91

### 3.5 Feature importance and SHAP analysis

**Figure 6:**
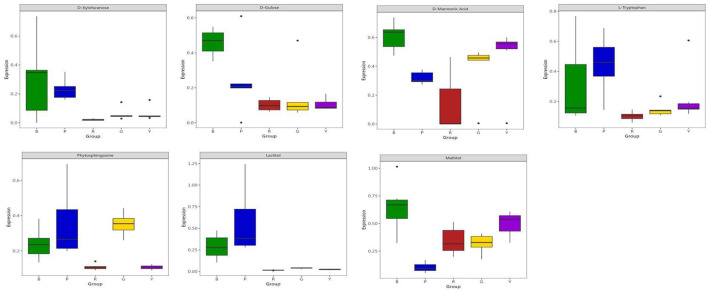
Box plot of the 7 feature metabolites in different pigmented rice varieties.

Figure 5E shows the feature importance of the selected 7 metabolites for prediction through machine learning, which revealed that phytosphingosine and lactitol were the top 2 features. Through SHAP analysis, the influence intensities of the import eigenvalue can be compared in order. In the present study, the 7 metabolites were ranked by the average SHAP value, which denoted the scale of the influence of each variable on the simulated export. As illustrated in Figures 5F, 6, phytosphingosine and lactitol contributed to the model discriminating the different pigmented rice varieties, which was similar to the feature importance outcomes. Moreover, the seven features contributing to the prediction varied among different pigmented rice varieties, a screening method that was also employed by Zhang et al. (21).

Phytosphingosine had a greater impact on the prediction of yellow-colored rice and green-colored rice than did the other three rice varieties. Lactitol and L-tryptophan had greater impacts on the prediction of red-colored rice than the other four rice varieties did (Figure 6). D-glucose and D-mannonic acid had greater impacts on the prediction of black-colored rice than did the other four rice varieties. Maltitol had a greater impact on the prediction of purple-colored rice than on that of the other four rice varieties (Figures 5F, 6). Moreover, coupling the feature importance value with the SHPA value examination can visually show the influence allocation for each sample, offering a probable justification for the forecast simulation and its dependence on metabolites in different pigmented rice varieties.

The long-chain base phytosphingosine is a component of sphingolipids and is present in yeast, plants and some mammalian tissues (31). Sphingosphingoid is the main component of plant biofilms and an important bioactive molecule in cells. It is involved in a variety of signal transduction pathways plays a vital role in plant growth, development and response to biotic and abiotic stresses. In the present study, it was found in pigmented rice, and it serves as an important differentially abundant metabolite.

Tryptophan (TRP) is converted into countless chemicals of biological significance, such as vitamins, and auxins (32). Fatchiyah et al. (33) used high-performance liquid chromatography (HPLC) to analyze L-tryptophan (Trp) in black rice samples and reported that Trp was the main precursor for the formation of phenolic compounds.

Soluble sugars contribute to many biological processes and structural constituents of cells (34). Kotamreddy et al. (35) found variations in glucose content in red, white and black rice via GC–MS in conjunction with metabolomics. Sugars and sugar alcohols play vital roles in the response of plants to salinity and drought stresses (36). By gas chromatography coupled with time-of-flight mass spectrometry (GC–TOF–MS), Kim et al. (37) detected three sugar alcohols in black and white rice. Figure 6 shows the boxplot profiles of the 7 features extracted from the differentially abundant metabolites in different pigmented rice varieties, which can well depict and distinguish the overall metabolomic profiles in pigmented rice. Compared with those of the other types, the levels of D-xylopyranose and D-fructose were significantly greater in black rice, indicating possible metabolic enrichment unique to black rice. D-mannonic acid was expressed at the highest level in red rice, while yellow rice also presented elevated levels, suggesting its potential involvement in oxidative stress or mannose metabolism. L-tryptophan and phosphoglycerate were predominantly elevated in purple rice, reflecting differences in amino acid and glycolytic metabolism. Notably, lactitol was almost exclusively expressed in purple rice, whereas the other types presented nearly zero levels. Similarly, maltitol levels were substantially higher in red rice and yellow rice.

The present study considered D-mannonic acid, maltitol and lactitol to be important differentially abundant metabolites, which is similar to the findings of the abovementioned reports (36, 37). Studies have shown that maltitol promotes the growth of beneficial gut bacteria, such as bifidobacteria and lactobacilli, and increases short-chain fatty acid production, enhancing gut health (38). Similarly, lactitol has been found to support the growth of these beneficial microbiota and increase the levels of SCFAs, such as butyrate and propionate, which are beneficial for gut function (39). Additionally, D-mannonic acid, a derivative of mannose metabolism, has been linked to antioxidant properties and may help modulate oxidative stress, although more direct studies are needed to fully elucidate its role in cellular protection. This finding also indicated that the key features of volatile metabolites in various colored rice varieties could be effectively extracted via GC–MS coupled with machine learning. Similar reports about features selected on the basis of metabolomics and machine learning were also published (16, 23). However, the present data and sample size were small, and the results should be expanded and validated in the future.

## 4 Conclusions

In summary, GC–MS-based metabolomics of different pigmented rice varieties was characterized, and 127 differentially abundant metabolites, which can favorably represent the majority of sample features, were screened. On the basis of metabolites with great multicollinearity above 0.8 and the chi-square test (20% feature numbers), only 7 metabolites were found to better represent the overall metabolites among the several colored rice varieties. The seven metabolites of the four machine learning models were further used for the classification of different pigmented rice varieties. The random forest model was the optimum for predicting classification, with an accuracy of 0.97. Moreover, SHAP analysis revealed that 7 metabolites can be used as potential markers for representing the metabolomic profiles. Overall, these results could provide insights into the distinctness of key gaseous metabolites in the five colored rice varieties. Machine learning approaches have proven to be rapid, useful tools for identifying key features of large data sets related to metabolomic analysis.

## Data Availability

The original contributions presented in the study are included in the article/Supplementary material, further inquiries can be directed to the corresponding authors.
