# Supplementary material for: Characterization and feature selection of volatile metabolites in Yangxian pigmented rice varieties through GC-MS and machine learning algorithms
Source: Front Nutr. 2025 May 20;12:1598875. doi: 10.3389/fnut.2025.1598875 (PMC12129771; doi:10.3389/fnut.2025.1598875)
Supplement: Supplementary file 1 [file Data_Sheet_1.pdf]

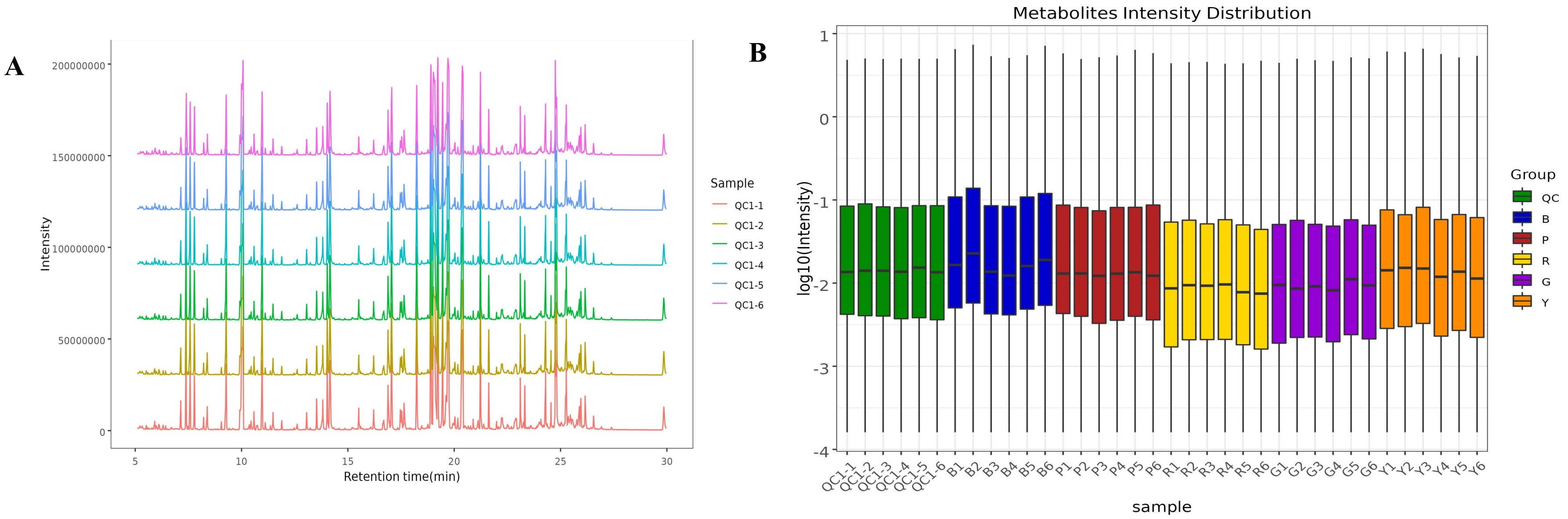

Fig. S1. The instrumental spectrum of QC and different pigmented rice. (A) total ion chromatogram of QC, and (B) metabolites intensity distribution of QC and pigmented rice.

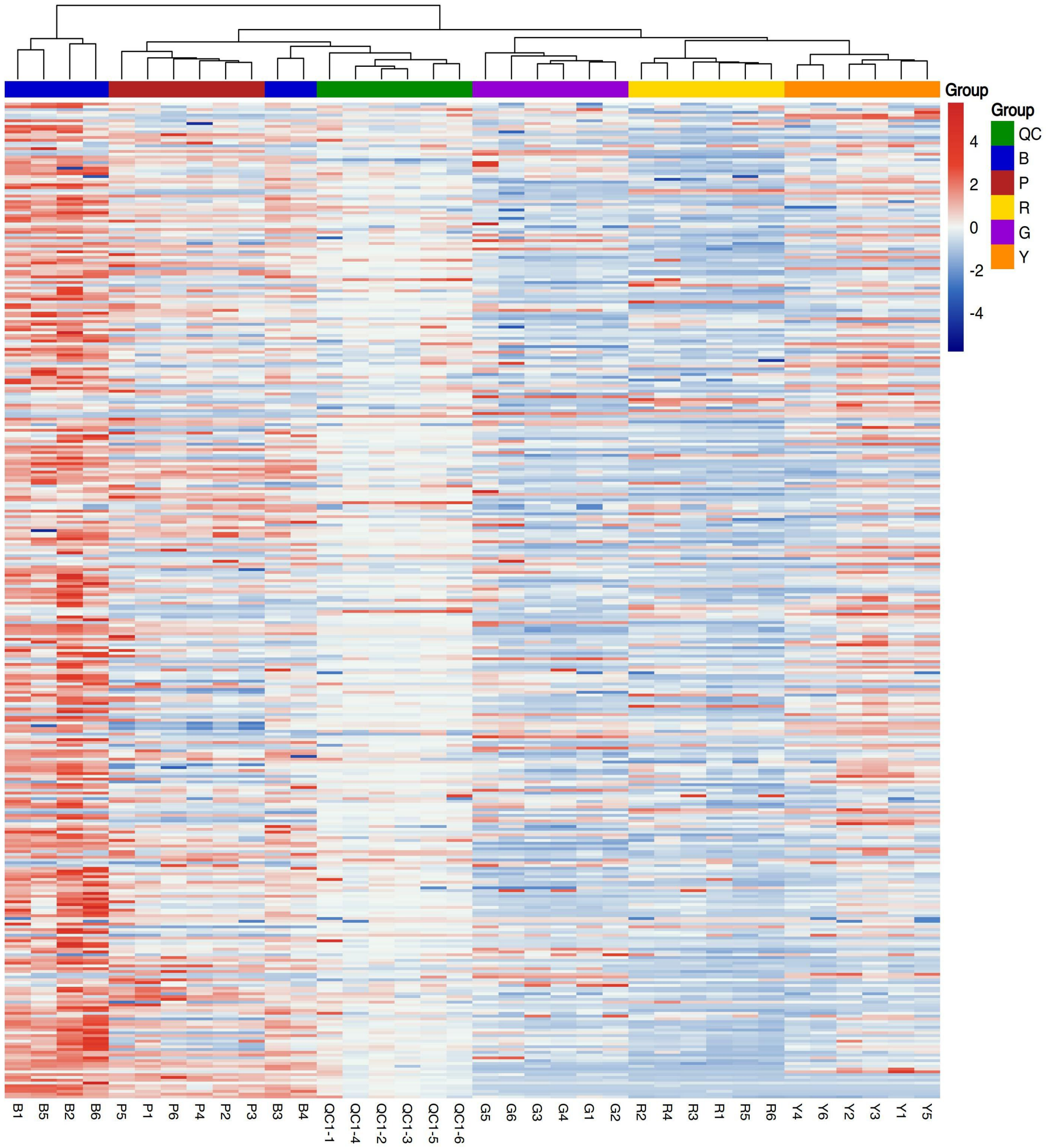

Fig. S2. Clustering heatmap of overall metabolites in QC and different pigmented rice

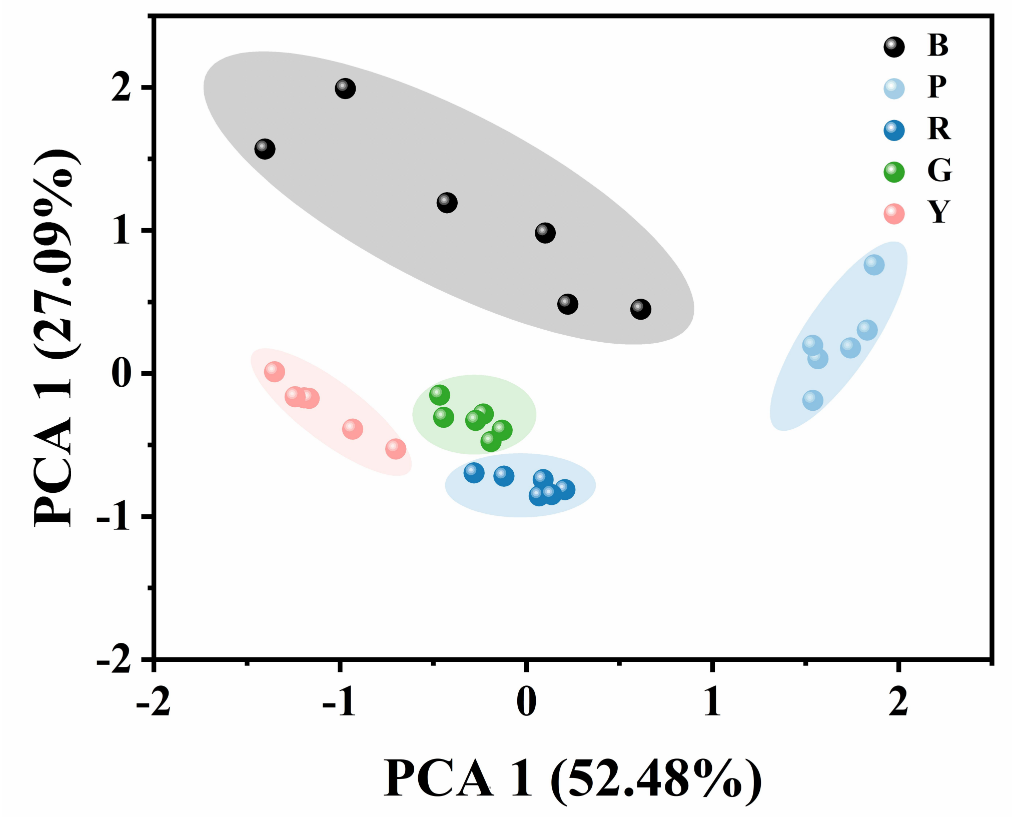

Fig. S3. PCA score plot of 127 differential metabolites in different pigmented rice.

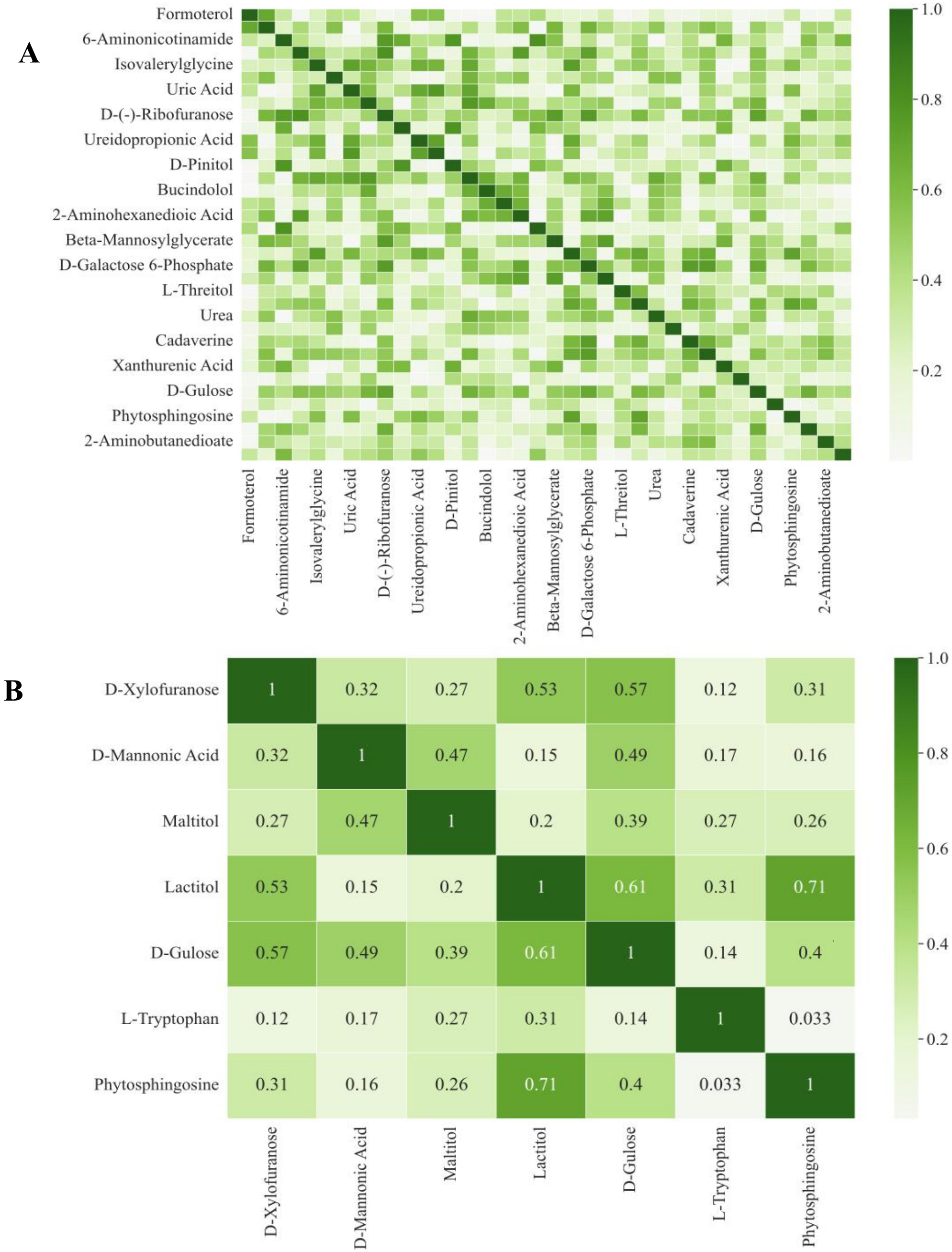

Fig. S4. Correlation coefficients of differential metabolites in different pigmented rice. (A) metabolites with great multicollinearity above 0.8, and (B) 20% feature numbers based on chi-squared test.
